# Perspectives of international multi-center clinical trials on traditional Chinese herbal medicine

**DOI:** 10.3389/fphar.2023.1195364

**Published:** 2023-05-18

**Authors:** Shan Wu, Chuanchi Wang, Dong Bai, Nanjie Chen, Jingqing Hu, Junhua Zhang

**Affiliations:** ^1^ Xin-Huangpu Joint Innovation Institute of Chinese Medicine, Guangzhou, China; ^2^ Institute of Basic Theory for Chinese Medicine, China Academy of Chinese Medicine Science, Beijing, China; ^3^ China Science and Technology Development Center of Chinese Medicine, Beijing, China; ^4^ Institute of Traditional Chinese Medicine, Tianjin University of Traditional Chinese Medicine, Tianjin, China

**Keywords:** traditional Chinese herbal medicine, multi-center, clinical trial, clinical value, clinical epidemiology

## Abstract

With the introduction of various subjects, such as clinical epidemiology and evidence-based medicine, the qualities and levels of Traditional Chinese Herbal Medicine (TCHM) in China improved substantially, and the processes of internationalization of Traditional Chinese Medicine (TCM) are further accelerated. Since, a variety of drug products in China have been approved for marketing in other countries, and approximately 10 products have submitted the IND application to FDA of United States, of which various Chinese herbal preparations such as compound Danshen dripping pills, Xingling granules, and HMPL-004 have been approved to be investigated in phase III clinical trials. In general, multi-center studies of TCHM are increasing with years, but most of the studies are performed in some certain country, and the actual international multi-center clinical trials are very rare. Number of SCI literatures on multi-center clinical trials of TCHM that published in the recent decade also showed increasing tendency with years, despite the evident reduction in the past 2 years due to the influence of COVID-19 pandemic. Of the multi-center clinical trials of TCHM that performed by mainland China and other oversees regions, except for Taiwan, China, nearly 70% were focused on classic Chinese medicinal formulae and Chinese patent medicine, while the other 30% were on dietary supplements and plant extracts. Facing the future, the “human experience” has attracted close attentions from researchers throughout the world. Effectively utilizing the historic “human experience” is an important method to vitalize potential of original scientific and technological resources of TCHM. Performing multi-center clinical trials with high qualities is still an essential method for TCHM in accessing the mainstream medicine market. In addition, it is also required to further improve the evaluation techniques and methods that not only meet the international standards but also meet the characteristics of TCHM. Furthermore, we should also focus on the TCHM specific clinical values and scientific reports.

## 1 Introduction

The unique theories of Traditional Chinese Medicine (TCM) and complexity of Chinese herbal compounds have made it quite difficult to evaluate the treatment efficacies of Traditional Chinese Herbal Medicine (TCHM) in studies, and the various indicators that difficult to be qualified also influenced the objective and accurate assessment of safety and effectiveness of TCHM (Shen et al., 2013). Clinical trial of drugs is an important process in drug development, and the quality could directly influence the safety and effectiveness of drugs after marketing. In this globally rampant pandemic of COVID-19, TCHM has been extensively used throughout the anti-pandemic processes, various TCHM that represented by Qing-Fei-Pai-Du Decoction, Huashibaidu Formula, Xuanfeibaidu Formula, Jin-Hua-Qing-Gan Granule, Lian-Hua-Qing-Wen Capsule, and Xuebijing injection have become important foreign-aid materials (Zhang et al., 2020), and have been approbated by various countries. In the past 4 decades, the introduction of subjects such as clinical epidemiology and evidence-based medicine has substantially improved the qualities and levels of clinical studies on TCHM, and a lot of high-quality high-level clinical studies on TCHM [including Investigational New Drugs (IND) not aiming for registration] have been performed successively, and a large group of TCHM have been registered for marketing in other countries, which have promoted the advancement of TCHM industry and the internationalization of TCHM.

## 2 Registration, marketing, and clinical trials of Chinese domestic TCHM overseas

According to the statistics of World Health Organization (WHO), TCM has been acknowledged by 29 countries and regions, including Australia, Canada, Austria, Singapore, Vietnam, by legislations, and 18 countries and regions have included TCHM in medical insurance. A lot of TCHM have been registered successively in the European Union (EU), Russia, Singapore, Cuba, and Vietnam.

The current 2004/24/EC decree on traditional herbal medicinal product (THMP) that issued by EU in 2004 provided a simplified registration procedure of THMP that had been used traditionally for long term but lacked evidence from modern studies, of which the “non-clinical” and “clinical” studies were derated from the application files of the Common Technical Document (CTD) of the products, while only literatures, evidence from experts, and reviews and reports on safety were required to demonstrate that the product had sufficient traditional use and safety, after which the product could be registered for marketing. Such measurements substantially reduced the difficulties in registration. This decree also encouraged the approval of THMP, including compound products of TCHM, to obtain EU market admittance for drugs (Qu et al., 2021). Currently, several varieties including Di’ao Xinxuekang capsule produced by the Chengdu Di’ao Pharmaceutical Group (2013, Netherland) (Wang, 2012), Danshen capsule produced by the Tasly Pharmaceutical Group Co. LTD. (2016, Netherland) (Zhang et al., 2017), Yufengningxin tablets produced by the Tong Ren Tang Group (2019, Netherland) (The Medicines Evaluation Board, 2019), and Banlangen granule produced by the Xiangxue-Cambridge International Research Center of Traditional Chinese Medicine (United Kingdom, 2015) (Medicines & Healthcare products Regulatory Agency, 2022) have been registered for marketing in EU.

TCHM could be registered as natural health products (NHPs) in Canada through the “traditional-application (also known as traditional efficacy application)” processes, for which the TCHM products of the formula, preparation processes, and indications being used for more than 50 years. Regarding the preparation of files for application, the files on treatment efficacies of the products are relatively flexible, while the safety and quality of the products are highly concerned. Since 2010, several TCHM products have acquired the marking qualification in countries other than China (Table 1). In 2008, the Fufang Danshen dripping pill and Chaihu dripping pill produced by the Tasly Group passed all the review and approval procedures by Health Canada as a Traditional Drug (Zhao et al., 2009). In 2016, the Danning tablet produced by the Shanghai Hutchison Pharmaceutical Co., LTD. was approved for marketing by the Natural and Non-prescription Health Products Directorate of Health Canada (Huang et al., 2018). In addition, the Juhong Tan Ke liquid produced by the Xiangxue Pharmaceutical Group (2012) and Antiviral Oral-Liquid (2016) were also approved by Health Canada (Xiangxue Pharmaceutical, 2021).

**TABLE 1:** Marketing of traditional Chinese herbal medicine overseas.

Serial No.	Name of TCHM	Applicant	Indication	Time and region of marketing	Notes
1	Huatuo Zaizao pill	Guangzhou Qixing Pharmaceutical Co., LTD.	Cerebrovascular and cardiovascular diseases	Russia, 2010	Permanent import drug license
2	Di’ao Xinxuekang capsule	Chengdu Institute of Biology & Di’ao Pharmaceutical Group	Coronary heart disease and angina pectoris	Netherland, 2012	
3	Tongxinluo capsule	Yiling Pharmaceutical Co., LTD.	Cerebrovascular and cardiovascular diseases	Vietnam, 2013	The first medicine included by medical insurance overseas
4	Antiviral oral-liquid	Guangzhou Xiangxue Pharmaceutical Co., LTD.	Anemopyretic cold, influenza	Canada, 2015	
5	Danshen capsule	Tasly Pharmaceutical Co., LTD.	Alleviating mild dysmenorrhea	Netherland, 2016	
6	Danning tablet	Shanghai Hutchison Pharmaceutical Co., LTD.	chronic cholecystitis, calculous cholecystitis	Canada, 2016	
7	Concentrated Danggui pill	Lanzhou Foci Pharmaceutical Co., LTD.	blood insufficient atrophy, irregular menstruation, and dysmenorrhea	Sweden, 2010, preliminary hearing for marketing	
8	Danning tablet	Shanghai Hutchison Pharmaceutical Co., LTD.	Obstructive jaundice	Canada, 2017	The first compound TCM with the “major functions” all approbated by regulatory authorities of European and American countries
9	Banlangen granule	Xiangxue-Cambridge International Research Center of Traditional Chinese Medicine, wholly-owned subsidiary corporation of Xiangxue Pharmaceutical Co., LTD.	Common cold	United Kingdom, 2017	
10	Lemai granule	Sichuan Chuanda Huaxi Pharmaceutical Co., LTD.	Acute or chronic cerebrovascular and cardiovascular diseases	Canada, 2017	The first Chinese patent medicine approved for marketing in Canada as medicine instead of healthcare product
11	Lian-Hua-Qing-Wen capsule	Yiling Pharmaceutical Co., LTD.	Influenza	Ukraine, 2020, registered as dietary supplement	

The Huatuo Zaizao pill produced by the Guangzhou Baiyunshan Qixing Pharmaceutical Co., LTD. is one of the confidential products in China, which has accessed 27 countries and regions including Russia, Canada, Australia, and Vietnam, and has been included in the medical insurance of Vietnam and Essential Medicine List of Russia. Huatuo Zaizao pill has already acquired the permanent drug registration certificate in Russia in 2010. In May 2012, the Qixing Pharmaceutical Group signed an “evidence-based clinical study agreement for treating cerebral stroke in rehabilitation stage by Huatuo Zaizao pill” with the Belarus National Scientific Practice Center of Cardiology, and jointly initiated the evidence-based clinical study of “Huotuo Zaizao pill.” In 2005, Xuezhikang was registered in Taiwan, China and Singapore as a prescription drug, and the clinical study results were published in Norway in 2006. In 2011, the clinical findings of Xuezhikang were included in the *European Guidelines for Blood Lipid Management*. Furthermore, the Juhong Tan Ke liquid produced by the Xiangxue Pharmaceutical Group was approved in Kenya (2013), and Xiaoerhuashi oral-liquid was approved for marketing by the Singapore Health Sciences Authority (2017). Lian-Hua-Qing-Wen Capsule was approved for marketing as “Chinese patent medicine,” “medicine,” “botanical medicine,” “natural health product,” “dietary supplement,” “modern botanical medicine,” and “natural medicine” in Hong Kong and Macao, China, Brazil, Indonesia, Canada, Mozambique, Romania, Thailand, Ecuador, Singapore, Laos, Kyrgyzstan, Philippines, Kuwait, Mauritius, Uganda, and Russia. The registration of Lian-Hua-Qing-Wen Capsule has also been initiated in the Middle East and Africa.

Substantially different from EU and other regions, TCHM could only apply for marketing in the United States through the new drug application (NDA) procedures, according to the *Guidance for Industry Botanical Drug Products* that issued by the Food and Drug Administration (FDA) of USA. In recent years, approximately 10 TCHM products submitted IND application to FDA (Table 2). The compound Danshen dripping pills produced by the Tasly Pharmaceutical Group Co., LTD., Xingling granules produced by the Xingling Sci-tech Pharmaceutical Co., LTD., and HMPL-004 produced by the Hutchison Whampoa Co., LTD. have been approved to be investigated in phase III clinical trials.

**TABLE 2:** Clinical trials of traditional Chinese herbal medicine in China.

Serial No.	Name of TCHM	Applicant	Indication	Status of clinical trial
1	Compound Danshen Dripping pill/Dantonic Capsule/T89	Tasly Pharmaceutical Co., LTD.	Angina pectoris	Phase III clinical trial was initiated in 2012, and completed in March 2016
2	Compound Danshen Dripping pill	Tasly Pharmaceutical Co., LTD.	Acute mountain sickness	Phase III clinical trial was initiated in July 2021
3	HMPL-004	Hutchison Whampoa Co., LTD.	Crohn’s disease, ulcerative colitis	Phase II clinical trial for ulcerative colitis was initiated in 2013. Phase III clinical trial was terminated in October 2014
4	Xuezhikang capsule/XueZhiKang	Beijing WBL Peking University Biotech Co., LTD.	Hyperlipidemia	Phase II clinical trial was completed in December 2012, and status of phase III clinical trial was unknown
5	Fuzhenghuayu tablet/Fuzheng Huayu	Shanghai Sundise Traditional Chinese Medicine Co., LTD.	Hepatic fibrosis following hepatitis C	Phase II clinical trial was completed in August 2013, and status of phase III clinical trial was unknown
6	Guizhifuling capsule/KYG0395	Jiangsu Kanion Pharmaceutical Co., LTD.	Essential dysmenorrhea	Phase IIb clinical trial was initiated in 2012, and completed in July 2015
7	Xingling granules	Shanghai Xingling Sci-tech Pharmaceutical Co., LTD.	Coronary heart disease and angina pectoris	Phase I and II trials were waived, status of phase III clinical trial was unknown
8	Weimaining capsule	Huayi Pharmaceutical Co., LTD.	Lung cancer	Phase I clinical trial was waived, status of phase II clinical trial was unknown
9	Kanglaite injection	Zhejiang Kanglaite Pharmaceutical Co., LTD.	Non-small cell pancreatic cancer	Unknown status
10	Kanglaite soft capsule/Kanglaite Gelcap	Zhejiang Kanglaite Pharmaceutical Co., LTD.	Prostate cancer	Phase II clinical trial was terminated in December 2013, the current status is unknown
11	Lian-Hua-Qing-Wen capsule	Yiling Pharmaceutical Co. LTD.	Unknown	Phase II clinical trial has been approved
12	Qishen Yiqi Dripping pill	Tasly Pharmaceutical Co. LTD.	Chronic heart failure	Phase II clinical trial initiated in June 2021
13	Yangzheng mixture	Shandong Buchang Pharmaceuticals Co., LTD.	Cancer	Phase IV clinical trial initiated in June 2021
14	Yuxuebi tablet	China Resources Sanjiu Medical & Pharmaceutical CO., LTD.	Ankylosing spondylitis	Subject recruitment of phase IV clinical trial has not initiated up to today
15	Shexiang Baoxin pill	Shanghai Hutchison Pharmaceutical Co., LTD.	Angina pectoris	Phase IV clinical trial initiated in January 2021

## 3 Advances in international multi-center clinical trials on TCHM

**FIGURE 1:**
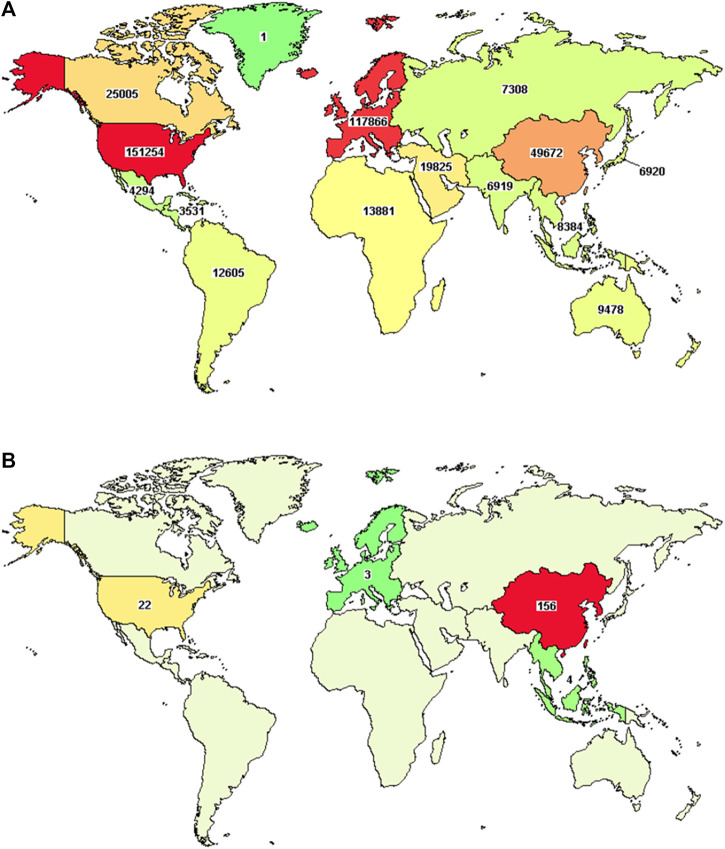
Distribution map of clinical trials that registered in United States clinical trial registry. [**(A)**, distribution of all clinical trials; **(B)**, distribution of clinical trials on TCHM]. The images are from publicly available data, the url: https://www.clinicaltrials.gov/ct2/results/map?term=Chinese+herbal+medicine&map=.

In general, international multi-center studies on TCHM are increasing with years in the recent decade, despite the fact that the total number is still limited. When using “Herbal medicine” as the keyword to search the United States Clinical Trial Registry, 212 clinical trials on TCHM were found registered in European or American countries (From January 1989 to April 2022). One hundred and fifty-six (73.58%) of the clinical trials were mainly from East Asia (including 104 from mainland China, 27 from Taiwan, China, and 15 from Hong Kong, China), 22 of the clinical trials were from North America (including 8 from New York, United States; 7 from California, United States; 3 from Connecticut, United States; and 3 from Maryland, United States), 4 of the clinical trials were from Southeast Asia, and 3 of the clinical trials were from Europe (Figure 1). Unfortunately, most of these studies were performed in some single countries, while the real international multi-center clinical trials were very rare. From the aspect of study status, 67 studies have completed as planned (31.60% completed), 39 studies are recruiting subjects (18.40% recruiting), 24 studies have not started the subject recruitment (11.32% Not yet recruiting), 6 studies terminated prematurely and subjects were not further treated or examined (2.83% Terminated), 3 studies are active and on-going (1.42% Active, not recruiting), 2 studies terminated prematurely but may be re-started again (0.94% Suspended), and 2 studies terminated before the recruitment of first subject (0.94% Withdrawn). From the phases of studies, 86 studies (40.57%) were phase II clinical trials, 34 studies (16.04%) were phase III clinical trials, 21 studies (9.91%) were phase I clinical trials, 21 studies (9.91%) were phase IV clinical trials, and 9 studies (4.25%) were early phase explorative trials.

**FIGURE 2:**
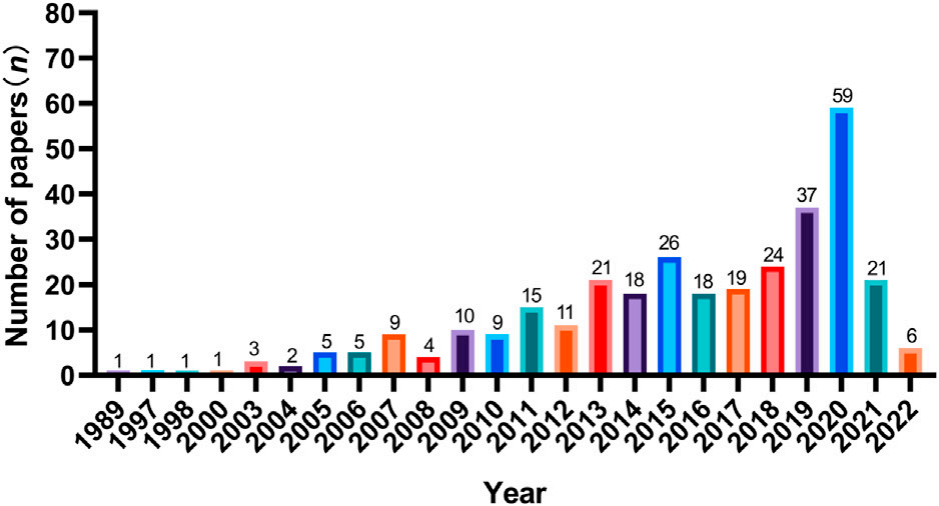
Years of publications of SCI articles of multi-center clinical trials on TCHM.

The number of SCI articles of multi-center clinical trials on TCHM is also increasing with years, and most of the studies were published in the recent 10 years. However, the number of studies was substantially lower in the recent 2 years, due to the influences of COVID-19 pandemic. When using “herbal medicine AND Randomized control AND multicenter” as the keywords to search the NewPubmed literature analysis system (pubmedplus.cn), 327 literatures were retrieved (From January 1989 to April 2022). Specifically, 4 studies (1.2%) were published in 1989–2000, 47 studies (14.4%) were published in 2001–2010, and 275 studies (84.1%) were published in 2011–2022. The number of studies published in 2020 was the highest (*n* = 59), which reduced to 21 in 2021, and further reduced to 6 in 2022 (Figure 2). China is the country with the most multi-center clinical trials on TCHM performed and most relevant SCI articles published in the world. The total number of SCI articles on multi-center clinical trials on TCHM in China was 263 (80.4%), followed by 5.2% in Japan, 2.8% in United States, 2.5% in Korea, and 2.1% in Pakistan. The five cities participated in most multi-center clinical trials on TCHM in China and overseas were Beijing, Shanghai, Guangzhou, Tianjin, and Chengdu, and Tokyo (Japan), Karachi (Pakistan), Fukuoka (Japan), Kyoto (Japan), Chiba (Japan), respectively. The subjects in these trials mainly with tumor, cardiovascular diseases, respiratory diseases, and gastrointestinal diseases.

**FIGURE 3:**
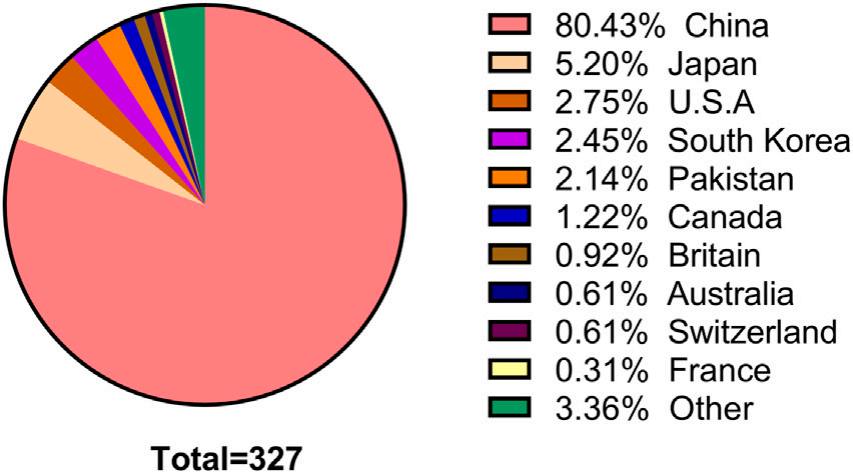
Countries of publications of SCI articles of multi-center clinical trials on TCHM.

Nearly 70% of multi-center clinical trials of TCHM performed in regions other than mainland China and Taiwan, China are on classic Chinese medicinal formulae and Chinese patent medicines, and the other 30% are mainly on dietary supplements or plant extracts. For instance, the following multi-center clinical trials were performed in Japan: Buzhong Yiqi decoction, Gegen decoction, and Xiaochaihu decoction for prevention and treatment of COVID-19; Wulingsan for treatment of glossalgia and prevention of recurrence of chronic subdural hematoma; Dahuang Mudan decoction for treatment of acute diverticulitis; Liujunzi decoction for the treatment of cervical carcinoma or endometrial carcinoma; Daikenchuto for the treatment of intestinal dysfunction after liver transplantation, as well as paralytic ileus after resection of colorectal carcinoma (CRC) or pancreatic carcinoma; Banxia Xiexin decoction for the treatment of CRC; Fangfeng Tongsheng granule for the treatment of obesity hypertension; Jinkui Shenqi pill for the treatment of chemotherapy induced peripheral neuropathy (neurotoxicity); and Goshuyuto for the treatment of headache. Furthermore, trial of Food Allergy Herbal Formula (FAHF-2) for the treatment of food allergy was performed in United States, and trials of Palm (Zonglv) pill for the treatment of chronic waist pain, as well as Ojeok-san (Wujisan) and Danggui Sini decoction for the treatment of cold hypersensitivity in the hands and feet were performed in Korea (Table 3; Figure 3).

**TABLE 3:** General characteristics of multi-center clinical trials on TCHM performed overseas (in regions other than mainland China and Taiwan, China) in the recent 15 years.

Trial	Country	Sample size		Disease of treatment	Intervention measurement		Treatment cycles	Primary outcome
		Treatment group	Control group		Treatment group	Control group		
Takao et al. (2021)	Japan	3,000	3,000	COVID-19	Oral intake of Hochu-ekki-to (Buzhong Yiqi decoction)	Placebo	8w	Positive results of PCR analysis of SARS-CoV-2RNA; number of patients with at least one of the following symptoms: fever, cough, expectoration, discomfort, and shortness of breath
Takayama S et al. (2020)	Japan	75	75	COVID-19	Routine treatment + Gegen decoction (kakkon-to:KT) 2.5 g and shosaikotokakikyosekko (ka-kikyo-sekko:SSKKS) 2.5 g	Routine treatment, using antipyretics, analgesics, or antitussives	2w	Days of alleviation of at least one of the following symptoms: fever, expectoration, cough, discomfort, and shortness of breath
Ayuse et al. (2020)	Japan	45	45	Glossalgia	Routine treatment + Wulingsan (Goreisan)	Routine treatment	12w	1) Assessment of VAS pain degree scale 2) Activity of salivary amylase (10 -200 kIU/L)
								3) Tongue examination (engorgement of dorsal lingual veins)
Ogawa-Ochiai et al. (2020)	Japan	85	85	Acute diverticulitis	Intravenous injection of antibiotics + oral intake of Dahuang Mudan decoction	Simple intravenous injection of antibiotics + placebo	10d	1) Success rate of diverticulitis treatment
								2) Hospital stay
								3) Improvement of inflammatory responses (c –reactive protein (CRP), white blood cells (WBC), and neutrophil count)
								4) Type of fever
								5) Days to initiation of food intake
								6) Recurrence rate (assessment at 1 year after registration)
								7) Incidence of adverse events
Jeong et al. (2020)	Korea	40	40	Mild neurocognitive disorder (mNCD)	Traditional herbal medicine Jujadokseo-hwan (consisted of 7 herbal components: aloe, angelica sinensis, pericarpium citri reticulatae, ginseng, liquorice, tuckahoe, and grifola)	Placebo	12w	1) Score of Seoul verbal learning test-elderly’s version (SVLT-E)
								2. SVLT-E, Rey complex figure test, Digit span test, Korean-Boston naming test, calculation ability, controlled oral word association test
Sung WS et al. (2019)	Korea	42	42	Chronic waist pain	Acupuncture + palm pill	Acupuncture	6w	1) Assessment of VAS pain degree scale
Ko et al. (2018)	Korea	30	30	Cold hypersensitivity in the hands and feet (CHHF)	Intake of herbal medicine Ojeok-sa​​n (Wujisan)	Placebo	8w	1) Assessment of visual analogue scale (VAS) score
								2) Mean changes in body skin temperature at particular acupoints, and total score of the Korean version of the WHO Quality of Life-BREF
Katayama et al. (2018)	Japan	92	88	Postoperative treatment of chronic subdural hematoma	Surgical treatment + oral intake of Wulingsan (goreisan)	Surgical treatment	12w	1) Recurrence rate of chronic subdural hematoma (CSDH)
								2) Hmatoma volume reduction rate shown by CT scanning
Ohnishi et al. (2017)	Japan	20	19	Cervical carcinoma or endometrial carcinoma	Standard treatment + oral intake of rikkunshito (Liujunzi decoction, consisted of atractylodes, ginseng, pinellia ternate, hoelen, violet, fructus aurantii, liquorice, and ginger)	Standard nursing	13d	Visual analogue scale (VAS) assessment, severity of nausea, and appetite
Ko et al. (2017)	Korea	33	33	Cold hypersensitivity in the hands (CHH)	Intake of Danggui Sini decoction	Placebo	6w	1) Visual analogue scale (VAS)
								2) Change of skin temperature of hands, Clinical global impression (CGI) scale, restoration rate of skin temperature of hands after cold stimulation, and total score of the Korean version of the WHO Quality of Life
Kaido et al. (2015)	Japan	55	55	Intestinal dysfunction after liver transplantation	Daikenchuto	Oral intake of placebo before meal	14d	1) Total oral or enteral caloric intake
								2) Abdominal distension determined using numeric rating scales (NRS)
								3) Abdominal pain determined using NRS.
Katsuno et al. (2016)	Japan	38	33	Colorectal carcinoma	Daikenchuto	Placebo	8d	X-ray evaluated gastrointestinal tract transit time or radiopaque markers, and the time to first flatus
Wang et al. (2015)	USA	46	22	Food allergy	Food Allergy Herbal Formula (FAHF-2; A preparation of 5 herbal medicines based on the TCHM Wuweiwan)	Placebo	24w	Incidence of adverse events and immunological parameters
Matsuda et al. (2015)	Japan	46	47	Colorectal carcinoma	Banxia Xiexin decoction	Placebo	2w	Safety, incidence of chemotherapy induced oral mucositis
Azushima et al. (2015)	Japan	54	52	Obesity hypertension	Fangfeng Tongsheng granule	Routine drug therapy	24w	Body weight, clinical blood pressure, dynamic blood pressure, brachial-ankle pulse wave velocity (baPWV), and incidence of adverse events
Oki et al. (2015)	Japan	89	93	Chemotherapy induced peripheral neuropathy (neurotoxicity)	Niuche Shenqi pill	Placebo	24w	Time of neuropathy treatment, incidence of adverse events
Kono et al. (2013)	Japan	44	45	Chemotherapy induced peripheral neuropathy (neurotoxicity)	Niuche Shenqi pill	Placebo	26w	Incidence of adverse events
Okada et al. (2013)	Japan	110	110	Paralytic ileus after resection of pancreatic carcinoma	Daikenchuto	Placebo	17d	Incidence of postoperative paralytic ileus lasting >72 h after surgery, time to first flatus after surgery, and gastrointestinal symptom rating scale score
Odaguchi et al. (2006)	Japan	28	25	Headache	Goshuyuto	Placebo	16w	Surface temperature of the thumb and toe, cutaneous blood flow, deep body temperature, the tissue oxygen saturation in the brain and the crural muscles, the hardness of the trapezius muscle, and the blood serotonin (5-HT) level

## 4 Thoughts and perspectives of international multi-center clinical trials on TCHM

The “human experience” of TCHM has attracted close attentions from researchers throughout the world, and effectively utilizing the historic “human experience” is an important method to vitalize potential of original scientific and technological resources of TCHM. In recent years, several countries and regions issued new drug development policies and laws with human use histories regarding the surveillance of traditional herbal medicine. Taking FAD as an example, the *Botanical Drug Development Guidance for Industry* was issued in December 2016 to provide guiding for pre-marketing reviewing of botanical drugs. The applicants are required to provide data of human experience when applying for phase I or phase II clinical trial, which could help providing surveillance requirements of pre-clinical and clinical studies. Countries or regions such as EU, Japan, and Korea also have special policies on reviewing, approving, and surveillance of traditional herbal medicines, Chinese prescription medicines, and prescriptions from Classic medicine books. “Human experience” is the summary of repeatable experience of treatments by TCHM that has certain regularities from long-term clinical practices. The historic human experience is mainly documented in ancient medical books, while the experience of using Chinese Patent Medicines is mainly from the use of preparations of medical settings and expert empirical prescriptions from prestigious physicians of TCM. It is not difficult to find that adhere to the guiding of clinical values, and establish the “three combinations” evidence system of TCHM reviewing based on TCM theories, human experience, and clinical trials will be the important innovation method for the advancement of TCHM to higher qualities and internationalization. How to summarize the involved scientific law and dig the implied scientific values of TCHM based on the thousand years’ historic human experience have profound strategic importance and important practical significances for improving the quality and efficiency of TCHM and promoting the processes of internationalization of TCHM. Therefore, we should not improperly belittle ourselves.

Performing multi-center clinical trials with high qualities is still the essential method for TCHM to access the mainstream international medicine markets. Since the implement of GCP, the overall quality of clinical trials of new drugs in China has improved substantially, despite that there are still several limitations (Yuan et al., 2021). Such as non-standard collection of human safety data, insufficient basis for researchers to judge the relationship between adverse events and trial drugs, and the inability to trace the main efficacy indicators such as scale in effectiveness data. To address these problems, targeted quality control measures should be formulated and principle of blind law should be strictly abide by. Encourage the use of modern technology, such as TCM equipment and other advanced tools for evaluation. The *14th Five Year Development Program for Pharmaceutical Industry* in Interpretation 2021 described supporting clinical study settings in China to actively participate and organize international multi-center clinical trials, and improve the internationalization levels of clinical trials (http://www.gov.cn/zhengce/2022-02/01/content_5671569.htm). Therefore, developing specific quality control measurements according to the characteristics of multi-center clinical trials when obeying the current GCP and ICH-GCP in China has important strategic significances for improving the development of new TCHM in China, as well as accessing the international mainstream medicine markets. The quality of TCM clinical trials depends on the design, especially the method of statistical data analysis. Therefore, in the process of trial design, considerations should be taken into account in how to collect research data, control test standardization and statistical analysis, so as to improve the quality of clinical trials. Besides, the clinical evaluation of TCM is a complex evaluation system. If we want to accurately screen the clinical trial indications of modern disease classification in early clinical research, and identify and evaluate the safety risk information number, we should reasonably use the information of the toxic action mechanism and target organs of TCM based on computer prediction. Network pharmacology in recent years has been widely used in the field of traditional Chinese medicine. The research methods based on network pharmacology can quickly identify the key medicinal components and targets of TCM, providing an important basis for the development and screening of TCM compounds in clinical practice. At the same time, it provides clearer guidance and direction for key safety signal monitoring in later large-scale clinical research.

It is necessary to further develop clinical evaluation techniques and methods not only meet the international standards but also meet the characteristics of TCHM. In recent years, the new techniques and methods of international clinical trials on drugs have been gradually applied in clinical trials of new drugs of TCHM, which showed the following advantages: 1) broke through the adaptive design of choosing the frequency-based statistical methods that has been long used in design and analysis in clinical trials; 2) enrichment strategy was developed to improve the accuracy of subject inclusion in clinical trials, and reduce the confounding factors (Yan et al., 2017); 3) master protocols and other high-efficient clinical trial design strategy, such as basket trial and umbrella trial (Guo et al., 2021), were used to improve the efficiency of clinical trials, shorten the duration of clinical trials, reduced and decrease of the costs of clinical trials. In 2015, China issued the formal announcement of the implement of ICH E17 guiding principle (National Medical Products Administration (NMPA), 2015).

Identifying the clinical position of drugs for treatment diseases that meet the characteristics of TCHM effects, and capable of leading to evident clinical values and benefits, and seeking, investigating, establishing, and designing scientific clinical efficacy evaluation tools and methods that been acknowledged by consensus to meet the clinical objectives and clinical positions will be an important study direction in the methodological field of clinical evaluation of new drugs of TCHM. Quality marker (Q-marker) was proposed by Liu C. X. et al. (2016) in response to the problem of TCM quality research. Since then, quality studies of TCM, in particular Q-marker studies were performed as to establish new research pattern. It will be of great significance to include Q-marker in TCM clinical trials in the future (Zhang T et al., 2018).

Additional attentions should be paid on TCHM specific clinical values and the scientific reports (Hu, 2021). In recent years, the relevant laws and regulations in China highly highlighted the TCHM specific clinical values. What are TCHM specific clinical values, and how to scientifically express them are very critical questions. It can be said that this is one of the questions related to the “life-gate” of high-quality development of TCHM industry. When facing the requirement for TCHM caused by deep aging, health issues caused by chronic non-communicable diseases, and threats from infectious diseases such as COVID-19, everyone are anticipating solutions from TCHM. However, comparing with the heated extolling of TCHM, the TCHM industry showed falling tendency in recent years, of which one important reason is that the specific clinical value has not been clearly described. TCHM has specific characteristics; however, acknowledged expression methods are required to describe the TCHM specific clinical values, which could help the mass to better understand. The clarification and description of clinical efficacy are the keys, instead of the mechanisms of effects. Artemisinin has already won the Nobel Prize, while the mechanisms of effects are still under investigation. The scientific evidence of clinical efficacy could be various, and the description of clinical values of TCHM should not only based on the results of randomized controlled trials, but also from other sources, such as case report, classic literatures, and records of clinical experience. The founder of evidence-based medicine, Gordon Guyatt, said that any empirical observations could be served as potential evidence, regardless of the systemic collection of it (Guyatt G et al., 2008). The focus of the innovation in the new TCHM reviewing and approval is the “three combinations” of clinical trials, human experience, and TCM theories. We speculated that systemically digging and scientifically evaluating the multidimensional evidence reaching the same study conclusion are worth encouraging. However, we need to further free our minds on how to promote the implement of relevant policies, which could free us from the limitations of randomized clinical trials.

In recent years, China has increased the investment on TCHM, and the internationalization of TCHM are now embracing the all-round, tridimensional, multi-level development, as a result of the promotion of “Belt and Road Initiative” construction and accelerated overseas layout of leading enterprises of TCHM. Despite the various challenges of integration of standards, policy barriers, and protection of intellectual property, TCHM internationalization has much to do and a long way to go, but still has a bright future.

## Abbreviations

CTD, Common Technical Document; CGI, Clinical Global Impression; FDA, Food and Drug Administration; GCP, Good Clinic Practice; ICH-GCP, Guideline for Good Clinical Practice of the International Conference on Harmonisation; ICH E17, ICH guideline E17 on general principles for planning and design of multi-regional clinical trials; IND, Investigational New Drug; NHPs, Natural Health Products; NDA, New Drug Application; TCM, Traditional Chinese Medicine; TCHM, Traditional Chinese Herbal Medicine; THMP, Traditional Herbal Medicinal P; WHO, World Health Organization.
